# Meta-analysis of the accuracy of transient elastography in measuring liver stiffness to diagnose esophageal varices in cirrhosis

**DOI:** 10.1097/MD.0000000000011368

**Published:** 2018-07-13

**Authors:** Fan Cheng, Hongyan Cao, Jinchun Liu, Lijun Jiang, Hongjuan Han, Yanbo Zhang, Dongxing Guo

**Affiliations:** a Department of Health Statistics, School of Public Health; b Department of Mathematics, School of Basic Medicine, Shanxi Medical University; c Department of Gastroenterology, The First Affiliated Hospital of Shanxi Medical University, Taiyuan, Shanxi, China; d Molecular Imaging Precision Medicine Collaborative Innovation Center, Shanxi Medical University,Taiyuan, Shanxi, China.

**Keywords:** esophageal varices, liver stiffness, meta-analysis, transient elastography

## Abstract

**Backgroud:**

To assess the diagnostic performance of transient elastography (TE) in detecting the presence and size of esophageal varices (EV) in cirrhotic patients.

**Methods:**

We searched PubMed, Web of Science, Wiley Online Library, Science Direct, China National Knowledge Infrastructure, WeiPu, WanFang database, and Baidu Scholar to identify studies that evaluated the diagnostic accuracy of TE in liver stiffness measurement, compared with esophagogastroduodenoscopy (EGD), for the detection of the presence and degree of EV in cirrhosis.

**Results:**

We included 32 studies in the presence of any EV (grade 1–3; n = 4082), 27 studies on substantial EV (grade 2–3; n = 5221) and 5 studies on large EV (grade 3). The pooled sensitivity, specificity, and diagnostic odds ratio (DOR) were 0.8 (95% CI, 0.78–0.86), 0.68 (95% CI, 0.62–0.74), and 10 (95% CI, 7–14) for any EV; 0.81 (95% CI, 0.77–0.85), 0.72 (95% CI, 0.66–0.77), and 11 (95% CI, 8–15) for substantial EV; and 0.92 (95% CI, 0.83–0.96), 0.78 (95% CI, 0.70–0.85), and 40 (95% CI, 15–107) for large EV. Subgroup analysis revealed that the heterogeneity among studies on any EV could potentially be explained by study location, proportion of Child A, and time interval between TE and EGD; for substantial EV, the proportion of Child A, etiology of cirrhosis, and the time interval between TE and EGD were important heterogeneity factors. Publication bias was found among studies evaluating diagnostic performance of TE for any EV.

**Conclusion:**

TE is a good tool for detecting the presence and degree of EV; however, in determination of the liver stiffness cutoff values means that TE is only cautiously used in clinical practice.

## 1 Introduction

Esophageal varices (EV) develops in nearly 50% of cirrhotic patients.^[1]^ Bleeding occurs in almost 25% of cirrhotic patients at 2 years,^[2]^ and the mortality risks from a first bleeding event is 20% to 35%.^[3]^ Thus, early diagnosis of cirrhosis is essential in preventing the development of disease and determining the immediate treatment. Esophagogastroduodenoscopy (EGD) is currently regarded as the gold standard for estimating the presence and degree of the EV in cirrhotic patients.^[1]^ However, EGD is invasive and probably increases the morbidity and mortality rate of complications.^[4]^ Other limitations, including patient discomfort and high medical costs, also cause a decline in patient compliance. The development of a noninvasive tool for evaluating the presence and extent of EV would be greatly helpful for the diagnosis and treatment of cirrhotic patients with EV.

Studies have suggested that liver stiffness values are potentially associated with EV.^[5]^ Liver stiffness can be measured by transient elastography (TE), which is a noninvasive method used to predict the presence and extent of EV in cirrhotic patients. TE provides high diagnostic accuracy for detecting the presence and severity of EV, and overcomes the limitations associated with therapeutic burden and patient discomfort.^[6]^ Recently, TE has been widely introduced into clinical practice; however, the predictive results of different studies have been shown to be inaccurate and unstable.^[5,7]^ Hence, this meta-analysis was conducted to systematically and comprehensively evaluate the diagnostic performance of TE for detection of the presence and size of the EV in cirrhotic patients.

## 2 Methods

### 2.1 Search strategy

We systematically conducted a literature search in PubMed, Web of Science, Wiley Online Library, Science Direct, China National Knowledge Infrastructure, WeiPu, and WanFang database to identify all studies conducted from January 1, 2006 to May 31, 2017 that evaluated the diagnostic accuracy of TE for the presence and size of EV. We used the medical subject heading search terms including “transient elastography,” “FibroScan,” “TE,” “FS,” “stiffness,” “esophageal varices,” and “cirrhosis.” No other search limitations were made. According to the inclusion and exclusion criteria described below, 2 workers preliminarily screened the titles and abstracts of literature independently to exclude irrelevant articles, and carefully evaluated the full text of the remaining articles. Next, additional articles were identified by manually searching the bibliographies of the key articles in Baidu Scholar. Finally, all eligible studies were included in this meta-analysis. During the retrieval process, any disagreements between the 2 researchers were resolved by discussion with a third author. This study followed the PRISMA guidelines.^[8]^

### 2.2 Selection criteria

The present meta-analysis included all prospective and retrospective full-text articles comparing the diagnostic performance of TE with EGD that satisfied the following inclusion criteria: EGD was used as a gold standard for the identification of EV in cirrhotic patients (grade 0: none; grade 1: small, straight varices; grade 2: medium-sized, enlarged tortuous varices occupying less than one-third of the lumen; grade 3: large-sized, coil-shaped varices occupying more than one-third of the lumen);^[9]^ liver stiffness was measured by TE to evaluate EV in cirrhotic patients older than 18 years, and was confirmed by liver biopsy or other clinical, biochemical, or imaging methods; sufficient information was available to precisely calculate true positive (TP), false positive (FP), true negative (TN), and false negative (FN) values of TE for the diagnosis of targeted disease; an optimal cutoff value was selected to maximize the sensitivity and specificity. There were no language restrictions imposed, but the included studies had sample sizes of at least 30.

We excluded studies that included patients younger than 18 years, patients with ascites, obesity, comorbid HIV, or liver transplantation that may have affected the TE result, and studies that provided unnecessary data such that no one 2 × 2 table could be created. When considering subsequent publications that were extensions of previously published cohorts, we only included the studies with the larger number of patients. For a number of studies with the same cohort but different results, we took the full approach to avoid missing important findings.

### 2.3 Data extraction

Using a data table formulated in advance, specific information was extracted independently by 2 researchers, including: basic characteristics: the first author, publication year, location of the study, study time period, and study design; patient characteristics: sample size, number of males, mean/medium age, body mass index, proportion of Child A, and etiology of cirrhosis; information regarding the index test (and reference standard): cutoff value, sensitivity, specificity (TP, FP, TN, and FN values could be computed), area under the receiver operating characteristics curve (AUROC), the presence or absence of blinding, and time interval between TE and EGD; information on study outcomes: EV grades, prevalence rates of EV, and Quality Assessment of Diagnostic Accuracy Studies-2 (QUADAS-2) scores.

### 2.4 Quality assessment

The methodological quality of studies was evaluated by 2 researchers using the QUADAS-2 questionnaire, which is a revised 14-item tool designed to evaluate the internal and external validity of diagnostic accuracy studies included in meta-analyses.^[10]^ Each term was judged as “yes” (one point) if it was reported, as “no” (zero points) if not reported, or as “unclear” (zero points) if the available information was not enough to allow a conclusion to be made. High-quality studies were defined as those with scores of 10 points or more. Disagreements between the 2 researchers were settled by discussion, with the final judgment made by a third researcher.

### 2.5 Outcome measures

The primary assessment was the diagnostic accuracy of TE in liver stiffness measurement (LSM) performed to detect the presence of EV (grade 1–3) and substantial EV (grade 2–3), compared with the gold standard of EGD. Considering potential heterogeneity among the studies, we conducted preplanned subgroup analyses based on 6 different variables to explore the sources of heterogeneity. The detection of large EV (grade 3) was the secondary assessment; we only reported the association between LSM detected by TE and large EV, without exploring the sources of heterogeneity.

### 2.6 Statistical analysis

The meta-analysis of diagnostic test accuracy was achieved by establishing a bivariate mixed effect model. All statistical analyses were performed with the Midas command in Stata 14.0 (StataCorp, College Station, TX).

#### 2.6.1 Indicators of diagnostic accuracy

Sensitivity (the proportion of those with the disease who have TP results) and specificity (the proportion of those without the disease who have TN results) are pooled summary statistics used to describe the accuracy of diagnostic tests. We calculated the positive likelihood ratio (PLR) as the sensitivity divided by the FP rate, and the negative likelihood ratio (NLR) as the FN rate divided by the specificity. We also calculated the diagnostic odds ratio (DOR), which is a comprehensive evaluation indicators defined as the ratio of the PLR to the NLR; the DOR indicates how much greater the possibility of contracting the disease is in subjects with a positive test result compared with subjects with a negative test result. We also plotted a summary ROC curve by considering the derived estimates of sensitivity, specificity, and respective variances, and the AUROC was regarded as an optimal measure of the diagnostic test to avoid the influence of different cutoff values. In addition, we assumed pretest probabilities of 25%, 50%, and 75%, and estimated the post-test probability with a positive or negative test result using Fagan plots.^[11]^

#### 2.6.2 Analysis of heterogeneity

The between-study heterogeneity of the pooled sensitivity and specificity was evaluated initially by Forest plots, then by statistical methods including the *Q* test and *I*^2^ statistic. A *P* value of <.10 or an *I*^2^ value of >50% was taken as an indicator of significant heterogeneity. If there was no threshold effect estimated by the Spearman correlation coefficient, sources of heterogeneity were explored using subgroup analysis based on the predefined characteristics of all studies.^[12,13]^

#### 2.6.3 Sensitivity analysis and publication bias

We also performed a sensitivity analysis to control for heterogeneity by excluding each of the studies one by one or excluding all studies with a methodological quality score below 10 points to verify the robustness of the diagnostic test accuracy.^[14]^ The publication bias was evaluated using the asymmetry test of the Deeks funnel plot, with *P* < .10 suggesting substantial asymmetry and significant publication bias.^[15]^

## 3 Results

### 3.1 Literature search

**Figure 1:**
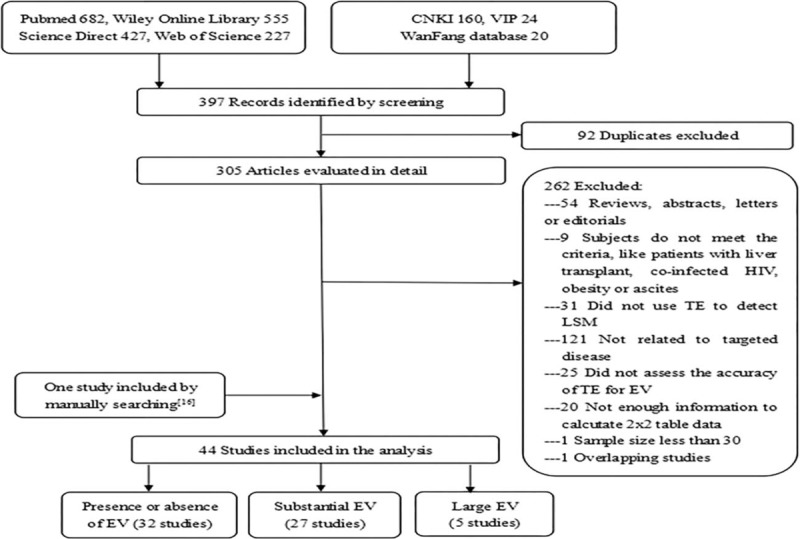
Flowchart of literature search and selection.

A total of 396 full-text articles were identified by screening. After removing duplicates, 305 remaining studies were evaluated in detail. Of these, 54 articles were excluded because they were reviews, abstracts, letters, or others; 152 studies were excluded because they did not relate to TE or EV; 45 studies were excluded because the diagnostic accuracy of TE for EV was not assessed, or there were insufficient information to calculate 2 × 2 tabular data; 11 studies were excluded due to inappropriate subjects, inadequate quantity, or overlapping records. A final total of 44 studies in which the diagnostic accuracy of TE for the detection of presence and size of EV was assessed were included in this meta-analysis. One included study^[16]^ was found by manual searching. Thirty-two included studies involved the diagnosis of the presence or absence of EV, 27 involved substantial EV, and 5 involved large EV (Fig. 1).

### 3.2 Characteristics and quality assessment of included studies

Liver stiffness cutoff values were 6.1 to 29.7 kPa for any EV, 12.2 to 48.0 kPa for substantial EV, and 17.7 to 34.6 kPa for large EV. Of the included 44 studies, 18 were performed in the European population, 21 in the Asian population (of which 16 were in China), 3 in the African population, and 2 in the North American population. The follow-up period in the earliest study started in 2002, and the study period of the latest study ended in 2016; the earliest study was a prospective study conducted in France, and the latest study was a retrospective study conducted in China. The follow-up duration of one study carried out in Romania was not reported. All articles published from 2006 to 2017 were prospective and retrospective studies. There were 7294 cirrhosis patients in total (mean age 54.26 years). Viral hepatitis was the leading cause of cirrhosis, with an etiology of HBV or HCV in only 13 studies. The etiology of cirrhosis in 2 studies was unknown. Details of the included studies are shown in Tables 1 and 2.

**Table 1:**
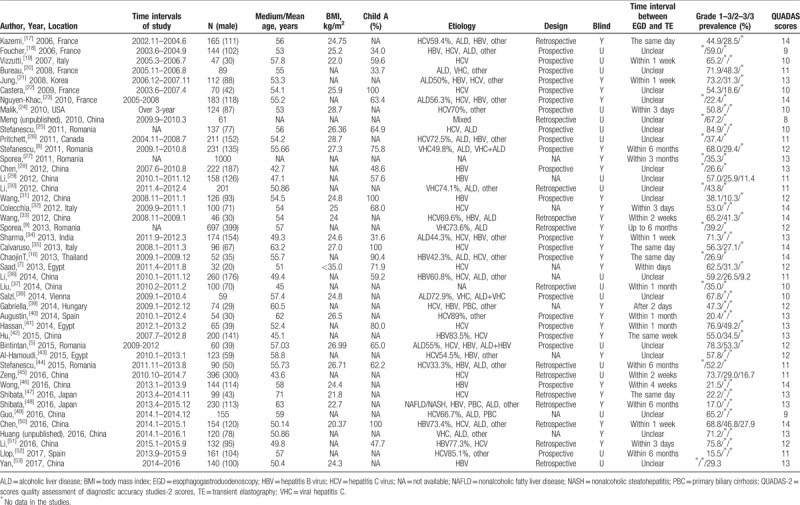
Basic information of included studies assessing the diagnostic accuracy of transient elastography.

**Table 2:**
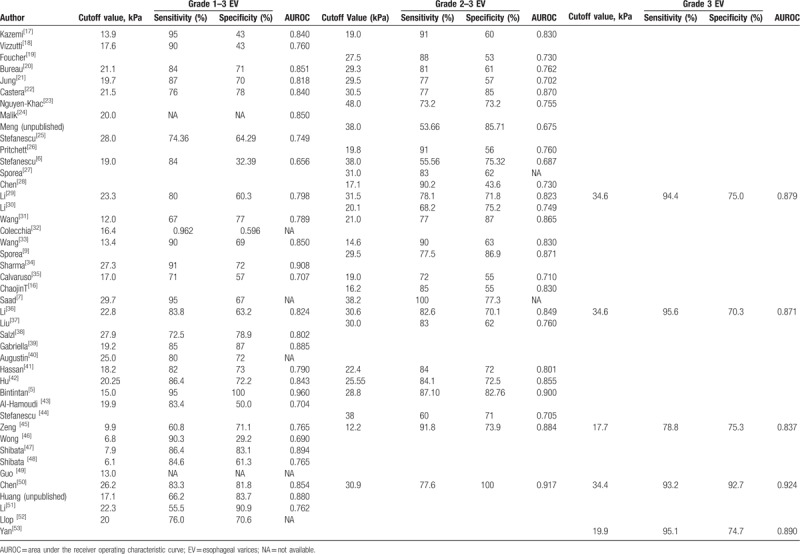
The results of evaluation of transient elastography for different stages of esophageal varices.

**Figure 2:**
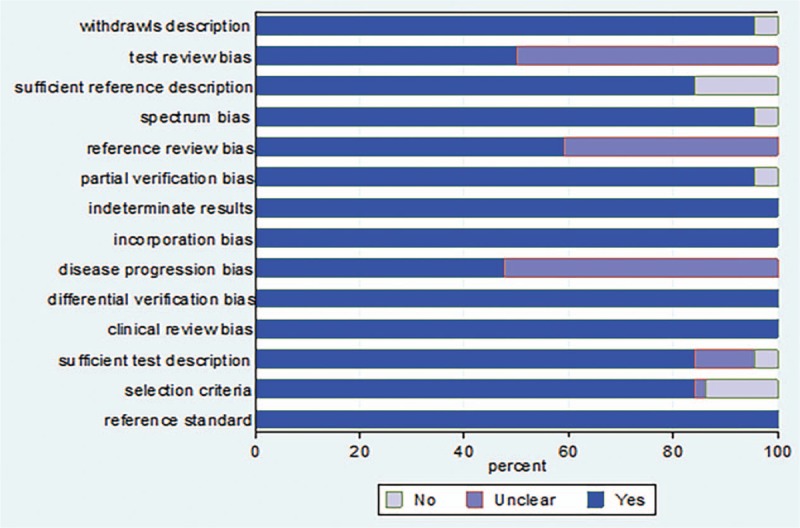
The graph of methodological quality of included studies.

The methodological quality assessment revealed that 3 of the 44 included studies achieved a score of 9 or lower; in general, the quality of all articles was moderate. On assessment of each QUADAS-2 item, 22 studies did not provide enough proof as to whether the TE results were interpreted by assessors blinded to the EGD results, while 18 studies did not state whether the EGD results were interpreted by assessors blinded to the TE results. Seven studies did not describe the gold standard and index tests in detail. The time interval between detection of LSM by TE and performance of EGD was not clearly noted in 23 studies. Seven studies did not describe inclusion and exclusion criteria of study subjects (Fig. 2).

### 3.3 Diagnosis of esophageal varices

Thirty-two studies (n = 4082) evaluated the diagnostic performance of TE for detecting the presence or absence of EV. Several significant pooled indicators were calculated; the pooled sensitivity was 82% (95% CI, 78–86%), the pooled specificity was 68% (95% CI, 62–74%), the PLR was 2.6 (95% CI, 2.1–3.1), and the NLR was 0.26 (95% CI, 0.21–0.33). The DOR was 10 (95% CI, 7–14), and the AUROC was 0.83 (95% CI, 0.79–0.86) (Table 3). However, significant heterogeneity was found among 32 studies (the *I*^2^ statistics were 86.31% and 86.60% for pooled sensitivity and specificity, respectively). Calculation of Spearman's correlation coefficient indicated that there were no threshold effects (coefficient = 0.24, *P = *.06).

**Table 3:**
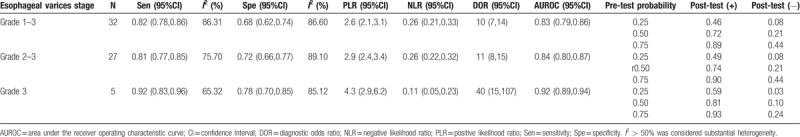
Summary indicators of the transient elastography for evaluating different Stages of esophageal varices.

Six subgroup analyses performed based on different variables indicated that the sources of heterogeneity may be explained by the study location, the time interval between TE and EGD, and the proportion of Child A (Table 4). Studies conducted in Asia (not in China) showed a superior diagnostic performance of TE compared with those conducted in China (DOR [95% CI] for Asia-China vs Asia-non-China: 8 [4–13] vs 22 [12–41]). The time interval between EGD and TE also influenced the predictive values of TE for targeted disease (DOR [95% CI] for TE and EGD performed within 1 week vs more than 1 week apart vs unclear: 16 [11–23] vs 6 [3–10] vs 8 [4–17]). Interestingly, DOR for the diagnosis of the presence of EV did not significantly differ between the subgroup with a proportion of Child A of more than 80% and the subgroup with no more than 80%, but the *I*^2^ values for the pooled sensitivity and specificity were 50% and 63%, respectively, indicating no significant heterogeneity.

**Table 4:**
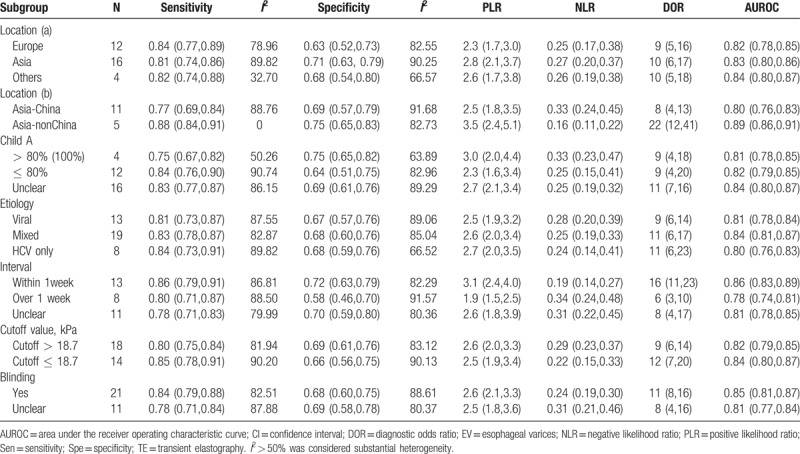
Subgroup analysis reporting the diagnostic accuracy of transient elastography for the liver stiffness measurement for evaluating the presence or absence of esophageal varices.

### 3.4 Diagnosis of substantial esophageal varices

Twenty-nine studies (n = 5221) were included in the analysis to assess the diagnostic performance of TE for substantial EV. The pooled sensitivity (95% CI) and pooled specificity (95% CI) were 81% (77%–85%) and 72% (66%–77%), respectively. The PLR (95% CI) and NLR (95% CI) were 2.9 (2.4–3.4) and 0.26 (0.22–0.32), respectively. The DOR was 11 (95% CI, 8–15). The AUROC was 0.84 (95% CI, 0.80–0.87) (Table 3). There was considerable heterogeneity observed in this analysis (the *I*^2^ values for the pooled sensitivity and specificity were 75.70 and 89.10, respectively), and the test results showed no evidence of a threshold effect (coefficient = 0.46, *P = *.22). Similarly, additional subgroup analyses were performed according to 6 factors (Table 5): 3 factors (proportion of Child A, etiology of cirrhosis, and time interval between TE and EGD) partially affected the heterogeneity between studies. The DOR of TE in study populations with more than 80% vs no more than 80% vs unclear proportion of Child A were 20 (4–109) vs 8 (6–11) vs 10 (7–15), respectively, indicating that a proportion of Child A of more than 80% was optimal. In subgroup analysis conducted by etiology of cirrhosis, the comparison of diagnostic performance of TE was made across patients with viral and mixed etiologies (DOR 15 and 10, respectively); in particular, for HCV only, the DOR was 19. We also categorized all included studies into 3 categories based on the time interval between TE and EGD. The results indicated that the DOR was better in the studies with a time interval between TE and EGD of within 1 week.

**Table 5:**
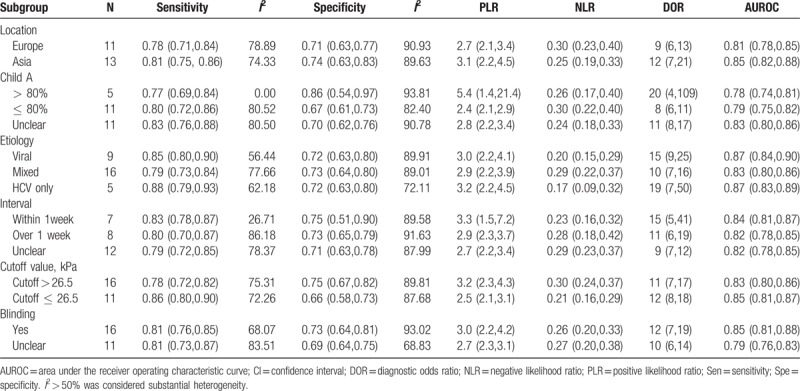
Subgroup analysis reporting the diagnostic accuracy of transient elastography for the liver stiffness measurement for detecting substantial esophageal varices.

### 3.5 Diagnosis of large esophageal varices

Five studies provided sufficient information on the diagnostic accuracy of TE for the diagnosis of large EV. In this meta-analysis, the summary sensitivity and specificity were 92% (95% CI, 83–96%) and 78% (95% CI, 70–85%), respectively. The PLR and NLR were 4.3 (95% CI, 2.9–6.2) and 0.11 (95% CI, 0.05–0.23), respectively. The DOR and the AUROC were 40 (95% CI, 15–107) and 0.92 (95% CI, 0.89–0.94), respectively (Table 3). There was significant heterogeneity for the summary sensitivity and specificity in this analysis. The testing of the threshold effect suggested that there may be a correlation between sensitivity and specificity (coefficient = 0.14, *P = *.02). Due to the small number of included studies, no further subgroup analysis was conducted to explore the sources of heterogeneity.

### 3.6 Sensitivity analyses and publication bias

Sensitivity analyses were performed by excluding 3 studies (Meng QQ, unpublished data, January 2010) with QUADAS-2 scores <10 points carried out in patients with any or substantial EV.^[19,49]^ For studies involving any EV, the pooled sensitivity, the pooled specificity, and the DOR were 0.82, 0.68, and 9, respectively, with an AUROC of 0.83, which suggested that the results did not differ substantially; for studies involving substantial EV, the pooled indicators corresponding to the summary sensitivity, specificity, DOR and AUROC were 0.82, 0.72, 11, and 0.84, respectively, which also indicated that there were no significant differences. A repeated analysis was also conducted after excluding each study sequentially, which showed that the results did not change significantly.

**Figure 3:**
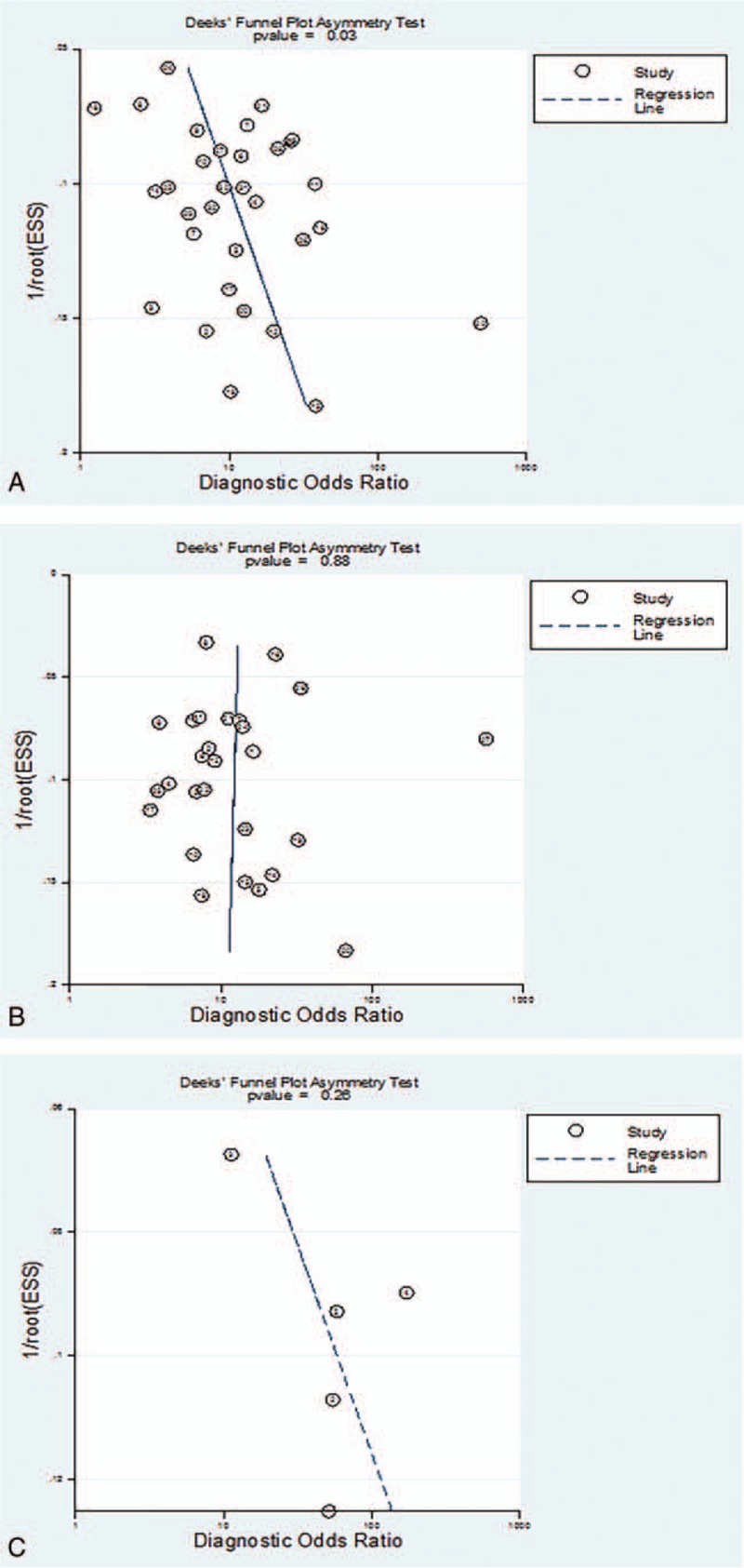
Deeks funnel plot of TE for the prediction of the presence and egree of EV. (A) TE detection of the presence of any EV. (B) TE detection of substantial EV. (C) TE detection of large EV. EV = esophageal varices, TE = transient elastography.

On implementing the asymmetry test of the Deeks funnel plot, a significant publication bias was found among studies evaluating TE for detecting the presence of any EV (*P = *.03) (Fig. 3A). There was no evidence of a great possibility of publication bias for TE for assessing substantial and large EV (*P = *.88 and *P = *.26, respectively) (Fig. 3B and C).

## 4 Discussion

In this meta-analysis, we identified 44 studies comparing TE (using FibroScan) with EGD for detecting the presence and degree of EV. Of these, 32 studies evaluated the diagnostic performance of TE for the presence or absence of EV (grade 1–3), 27 studies for substantial EV (grade 2–3), and 5 studies for large EV (grade 3). Calculating all summary indicators led to several important discoveries. First, TE had a relatively high diagnostic accuracy for assessing the presence of any EV, substantial EV, and large EV, with a summary AUROC of 0.83, 0.84, and 0.92, respectively. Second, the pooled sensitivity and specificity corresponding to the presence of any EV, substantial EV, and large EV were 0.82 and 0.68, 0.81 and 0.72, 0.92, and 0.78, respectively, indicating that the estimates of pooled sensitivity and specificity were considered to be good. In particular, the results for detecting large EV were almost excellent. However, the summary specificity for assessing any EV was not satisfactory. Third, TE showed a high DOR of 40 in identifying large EV; however, the DORs for any and substantial EV were less than ideal.

Our results suggest that TE could be used for diagnosing the presence or absence of EV. When pre-test probability equaled 50%, the probability of correctly diagnosing the presence of EV was 72% following a positive result. For later stage EV, the values were more convincing; the probabilities of correctly diagnosing substantial and large EV following a positive test result were 74% and 81%, respectively. If the test results were negative, there might still have been the presence of any EV present in 21% of patients, and large EV in 10%. When there was a high pre-test probability of 75%, then the probability of an accurate diagnosis following a positive test result was more than 90% for all EV stages, but an incorrect diagnosis would still be possible in 24% to 44% of patients with a negative test result.

We observed considerable heterogeneity among studies assessing the diagnostic accuracy of TE for detecting the presence or absence of EV. To determine the source of heterogeneity, all studies were divided into 3 subgroups based on the study location (European, Asian, and others). However, there was no obvious difference in the diagnostic performance between subgroups. Furthermore, all studies performed in Asia were divided into 2 groups (non-Chinese and Chinese), as significant heterogeneity was found among all Asian studies. The DOR for detecting EV in Asian-non-Chinese studies was more than twice as high as that in Chinese studies, and the I^2^ value for sensitivity was 0%, indicating that heterogeneity did not exist; this suggests that the heterogeneity of the studies may be explained by the different population groups. Although the DOR for identifying EV was 16 in studies in which the detection of EV was performed by TE within 1 week of EGD, and the DOR was 6 in studies where TE and EGD were performed more than1 week apart, the heterogeneity of the pooled sensitivity and specificity was still very significant. This observed difference is possibly associated with differences in indicators of the body checked by TE on different days, as the situation of patients is dictated by the severity of disease and the clinical setting.^[54]^ Therefore, the effect may have been better if all studies were divided into 2 subgroups: studies with EV detection via TE and EGD performed on the same day, and those that reported a time interval between TE and EGD. Although there was no obvious difference between the DOR values on the basis of the proportion of Child A, there was a reduction in variation of the pooled sensitivity and specificity. That is, the proportion of Child A might be the origin of heterogeneity; however, additional exploration in future studies is required to confirm this.

There was also significant heterogeneity among included studies evaluating substantial EV. The source of heterogeneity was explored according to the proportion of Child A, etiology of cirrhosis, and the time interval between TE and EGD. Within each subgroup, we considered that the DORs for evaluating substantial EV of different levels of variables were comparable, and that the *I*^2^ value for sensitivity or specificity was decreased. Thus, the variation in diagnostic performance of TE for substantial EV was potentially identified based on the proportion of Child A, etiology of cirrhosis, and time interval between TE and EGD; however, this result also should be considered with caution.

Although our meta-analysis is not the first to evaluate the diagnostic performance of TE for detecting the presence and degree of EV, we included more than twice the number of studies included in 4 previous meta-analyses published on the same subject.^[55–58]^ Li et al^[55]^ assessed the diagnostic accuracy of TE for detecting substantial EV in 20 included studies, and reported that summary sensitivity, specificity, PLR, NLR, DOR, and AUROC were 0.81, 0.71, 2.63, 0.27, 10.3, and 0.83 respectively, indicating that all pooled indicators were similar to ours. Pu et al,^[56]^ Qu et al,^[57]^ and Shi et al^[58]^ analyzed a total of 13, 20, and 12 studies, respectively, regarding the predictive accuracy of TE for detecting the presence of any and substantial EV, and reported results consistent with our research. Compared with other studies (including ours), the values of pooled specificity in the analysis by Shi et al^[58]^ were low (0.53 and 0.59 for any and substantial EV, respectively).

The first strength of our meta-analysis was the comprehensive assessment of the diagnostic performance of LSM measured by TE techniques for identifying the presence and size of EV. The association between LSM measured by TE and varying degrees of EV could be thoroughly analyzed by extracting a large amount of basic information. Most importantly, this is the first such review of the predictive performance of TE for detecting large EV; large EV carry a high risk of bleeding and mortality,^[2,3]^ which has a crucial impact on clinical practice and therapeutic decisions. Second, we exhaustively and vigorously searched numerous databases without language restrictions, resulting in the inclusion of a large number of studies in the final review. Furthermore, the latest publication year of studies included in this analysis was 2017,^[52,53]^ which makes the evaluation results more reliable and applicable to actual clinical practice. Third, as both sensitivity and specificity were independent of cutoff value, multiple subgroup analyses were carried out based on different variables to explore the sources of heterogeneity; we were able to potentially identify that the differences in diagnostic accuracy of TE for detecting the presence or absence of EV were mainly based on study location, time interval between TE and EGD, and proportion of Child A. For substantial EV, the potential heterogeneity primarily derived from the proportion of Child A, etiology of cirrhosis, and the time interval between TE and EGD.

Several limitations in our meta-analysis must be acknowledged. First, the limited number of included studies and the differences in TE cutoff values in each study made it difficult to define an optimal diagnostic threshold for accurate prediction of any and substantial EV. Second, assessment of methodological quality found that most studies provided insufficient information on whether the TE results were assessed by evaluators blinded to the EGD results, or vice versa, creating the risk of review bias. Furthermore, many studies did not state the time interval between TE and EGD, resulting in a risk of disease progression bias. Third, there was a significant publication bias in studies evaluating the accuracy of TE in detecting the presence of any EV. That is, the reproducibility and accuracy of the study conclusions would be limited because of negative results. We attempted to review all relevant research, including non-English language publications and postgraduate theses, to minimize the effect of publication bias (Meng QQ, unpublished data, January 2010; Huang LL, unpublished data, June 2016). Fourth, subgroup analyses that compared the observed differences in DOR values between several subgroups were conducted to explain the potential origins of heterogeneity; the decrease in *I*^2^ values of the pooled sensitivity and specificity indicate that the present results should be interpreted cautiously.

In conclusion, this meta-analysis found that TE has a high degree of predictive accuracy for identification of the presence and size of EV in patients with liver cirrhosis, as compared with EGD. In the field of liver disease, this available and noninvasive TE technique represents a major advance; however, TE has not yet displaced EGD as the method of choice for diagnosing EV. Additional high-quality studies and more advanced data analysis techniques are required to further prove the performance of TE.

## Author contributions

**Conceptualization:** Jinchun Liu, Lijun Jiang.

**Data curation:** Fan Cheng.

**Formal analysis:** Fan Cheng.

**Methodology:** Hongyan Cao.

**Project administration:** Yanbo Zhang, Dongxing Guo.

**Software:** Hongjuan Han.

**Supervision:** Yanbo Zhang, Dongxing Guo.

**Validation:** Hongyan Cao.

**Visualization:** Hongjuan Han.

**Writing – original draft:** Fan Cheng.
